# Mapping knowledge landscapes and emerging trends of the biomarkers in melanoma: a bibliometric analysis from 2004 to 2022

**DOI:** 10.3389/fonc.2023.1181164

**Published:** 2023-06-23

**Authors:** Yantong Wan, Junyi Shen, Yinghao Hong, Jinghua Liu, Tieliu Shi, Junwei Cai

**Affiliations:** ^1^ Guangdong Provincial Key Laboratory of Proteomics, Department of Pathophysiology, School of Basic Medical Sciences, Southern Medical University, Guangzhou, China; ^2^ The Second School of Clinical Medicine, Southern Medical University, Guangzhou, China; ^3^ The Center for Bioinformatics and Computational Biology, Shanghai Key Laboratory of Regulatory Biology, The Institute of Biomedical Sciences and School of Life Sciences, East China Normal University, Shanghai, China; ^4^ Beijing Advanced Innovation Center for Big Data-Based Precision Medicine, Beihang University & Capital Medical University, Beijing, China

**Keywords:** melanoma, VOSviewer, CiteSpace, biomarker, visual analysis

## Abstract

**Background:**

Melanoma is a skin tumor with a high mortality rate, and early diagnosis and effective treatment are the key to reduce its mortality rate. Therefore, more and more attention has been paid for biomarker identification for early diagnosis, prognosis prediction and prognosis evaluation of melanoma. However, there is still a lack of a report that comprehensively and objectively evaluates the research status of melanoma biomarkers. Therefore, this study aims to intuitively analyze the research status and trend of melanoma biomarkers through the methods of bibliometrics and knowledge graph.

**Objective:**

This study uses bibliometrics to analyze research in biomarkers in melanoma, summarize the field’s history and current status of research, and predict future research directions.

**Method:**

Articles and Reviews related to melanoma biomarkers were retrieved by using Web of Science core collection subject search. Bibliometric analysis was performed in Excel 365, CiteSpace, VOSviewer and Bibliometrix (R-Tool of R-Studio).

**Result:**

A total of 5584 documents from 2004 to 2022 were included in the bibliometric analysis. The results show that the number of publications and the frequency of citations in this field are increasing year by year, and the frequency of citations has increased rapidly after 2018. The United States is the most productive and influential country in this field, with the largest number of publications and institutions with high citation frequency. Caroline Robert, F. Stephen Hodi, Suzanne L. Topalian and others are authoritative authors in this field, and The New England Journal of Medicine, Journal of Clinical Oncology and Clinical Cancer Research are the most authoritative journals in this field. Biomarkers related to the diagnosis, treatment and prognosis of melanoma are hot topics and cutting-edge hotspots in this field.

**Conclusion:**

For the first time, this study used the bibliometric method to visualize the research in the field of melanoma biomarkers, revealing the trends and frontiers of melanoma biomarkers research, which provides a useful reference for scholars to find key research issues and partners.

## 1 Introduction

Melanoma is a malignant tumor derived from melanocytes, which mainly occurs in human skin, mucous membranes, conjunctiva, extremities and other parts (1, 2). As a rare disease, melanoma accounts for only 4% of skin cancer cases, but it has a very high fatality rate, accounting for 75% of all skin cancer deaths (3). In the past 30 years, the incidence of melanoma worldwide has increased steadily (4). In the United States alone, an estimated 99,780 new cases of cutaneous melanoma will be diagnosed and 7,650 deaths will occur in 2022 (5). The pathogenesis of melanoma is closely related to external factors, and the most important risk factor is ultraviolet radiation (6). Evidences show that a large number of UV-characteristic mutations, such as C→T and G→T, can be observed in melanoma cells (2, 7). In addition, ultraviolet rays can also suppress the immune system locally or systemically, creating conditions for immune evasion of cancer cells (1). Other risk factors associated with melanoma include the number of moles, age, and family history of skin cancer (1, 8, 9). In terms of genetics, the onset of melanoma is closely related to chromosomal aberrations and gene mutations in melanocytes (10, 11). Studies have reported that the loss of the tumor suppressor gene p16 is closely related to the occurrence of sporadic and familial melanoma (10). In addition, mutations in genes such as cyclin-dependent kinase inhibitor 2A (CDKN2A) and cyclin-dependent kinase 4 (CDK4) have been shown to be the most common genetic variants in familial melanoma (10).

Mucosal melanoma distributed in the rectum, eyes, mouth, and nasopharynx is usually difficult to detect in the early stage, and it has a high degree of malignancy and a poor prognosis as the disease progresses (12). Therefore, it is important to find a method that can assist in the early detection and treatment of melanoma. Biomarkers are tumor or host-related factors that correlate with tumor biological behavior and patient prognosis (13). In recent years, with the in-depth researches on the genetic basis and molecular mechanism of melanoma, the application value of biomarker in the diagnosis and treatment of melanoma has received more and more attention (14). In terms of diagnosis, as antibodies to melanoma antigens, Melan-A and MATT-1 are the most widely used biomarkers for the diagnosis of melanoma, with extremely high sensitivity (3, 14, 15). In addition, Biomarkers such as S100 protein, micropthlamia transcription factor (MITF), tyrosinase and SOX10 are also widely used in the diagnosis of melanoma (16–18). In terms of treatment, melanocyte proliferation markers can be used to assess the cycle activity of diseased cells to clarify the degree of malignancy of the tumor (19). Ki-67, phosphohistone H3 (PHH3), etc. are common proliferation markers, which can be used clinically to evaluate the therapeutic effect of melanoma (20, 21). Notably, serological markers such as lactate dehydrogenase (LDH) can also be used for the assessment of melanoma prognosis (16). In addition, related Biomarkers have also been applied to the evaluation of melanoma treatment effects. For example, blocking PD-1/PD-L1 is an attractive cancer immunotherapy strategy, and PD-L1 immunohistochemistry is currently widely used to predict the efficacy of melanoma treatment response (22–24).

Bibliometric analysis is a method of qualitative and quantitative review and analysis of research in a specific research field within a specific time period using mathematical and statistical methods (25). This method can focus on countries, institutions, journals, authors and keywords related to research in a specific field, providing readers with objective field development trends and cutting-edge hotspots (26, 27). Bibliometric analysis has been applied in many fields closely related to melanoma biomarker, including immune checkpoint blockade, uveal melanoma, anti-PD-1/PD-L1 cancer therapy, etc (28–30). Although the research on melanoma-related biomarkers has developed rapidly in the past two decades, there is still a lack of bibliometric analysis of the latest melanoma-related biomarkers. Therefore, this study aims to analyze the overall situation of melanoma-related Biomarker research through two bibliometric software, VOSviewer and CiteSpace, and determine the research trends and frontier hotspots in the past two decades, so as to help researchers understand the corresponding fields and find cooperation partners for reference.

## 2 Method

### 2.1 Data collection

The data for the econometric analysis of this study came from the Web of Science Core Collection (WOSCC). WOSCC is a comprehensive, standardized set of databases widely used in academia (31). The search set used in this study is “TS=(“Melanoma” OR “Melanomas” OR “Malignant Melanoma” OR “Malignant Melanomas” OR “Melanoma, Malignant” OR “Melanomas, Malignant”)” AND TS=(“Biomarkers” OR “Marker, Biological” OR “Biological Marker” OR “Marker, Biological” OR “Biological Markers” OR “Biological Markers” OR “Markers, Biological” OR “Biomarker” OR “Markers, Biological” OR “Markers, Biological” OR “Immune Markers” OR “Markers, Immune” OR “Marker, Immunologic” OR “Immunologic Markers” OR “Immune Marker” OR “Marker, Immune” OR “Immunologic Marker” OR “Serum Markers” OR “Markers, Serum” OR “Marker, Serum” OR “Serum Marker” OR “Surrogate Endpoints” OR “Endpoints, Surrogate” OR “Surrogate End Point” OR “End Point, Surrogate” OR “Surrogate End Points” OR “End Points, Surrogate” OR “Surrogate Endpoint” OR “Endpoint, Surrogate” OR “Markers, Clinical” OR “Clinical Markers” OR “Clinical Markers” OR “Marker, Clinical” OR “Viral Markers” OR “Markers, Viral” OR “Viral Markers” OR “Marker, Viral” OR “Biochemical Marker” OR “Markers, Biochemical” OR “Marker, Biochemical” OR “Biochemical Markers” OR “Markers, Laboratory” OR “Laboratory Markers” OR “Laboratory Marker” OR “Marker, Laboratory” OR “Marker, Laboratory” OR “Markers, Surrogate” OR “Marker, Surrogate” OR “Surrogate Marker”). The search period was limited from January 1, 2004 to September 17, 2022. Only “Article” and “Review” were selected for the article type, and the language was limited to English. Finally, 5584 documents were obtained. Search on WOSCC according to the above formula, and the results are exported as plain text documents in txt and csv formats. In order to prevent data bias caused by database updates, the literature search was completed on September 17, 2022.

### 2.2 Data analysis and visualization

CiteSpace, developed by Chaomei Chen, is currently the most widely used bibliometric analysis software (32). We used CiteSpace 6.1.R2 Advanced to visualize and analyze country distribution and collaboration, institution distribution, discipline regional distribution, keyword timeline map, literature explosion, etc. VOSviewer was developed by Nees Jan van Eck et al. It is mainly used for bibliometric network graph analysis (33). We used VOSviewer 1.6.18 to visually analyze country distribution, institution distribution, author distribution, keyword distribution, etc. In addition, we used Bibliometrix (R-Tool of R-Studio) (34) to visually analyze the distribution of countries, references and keywords, and used Microsoft Excel 365 to display the trend of publication and citation over the years. All primary data used in this study were obtained from public databases and therefore did not require ethical review.

## 3 Result

### 3.1 Annual publications and citation trends

**Figure 1:**
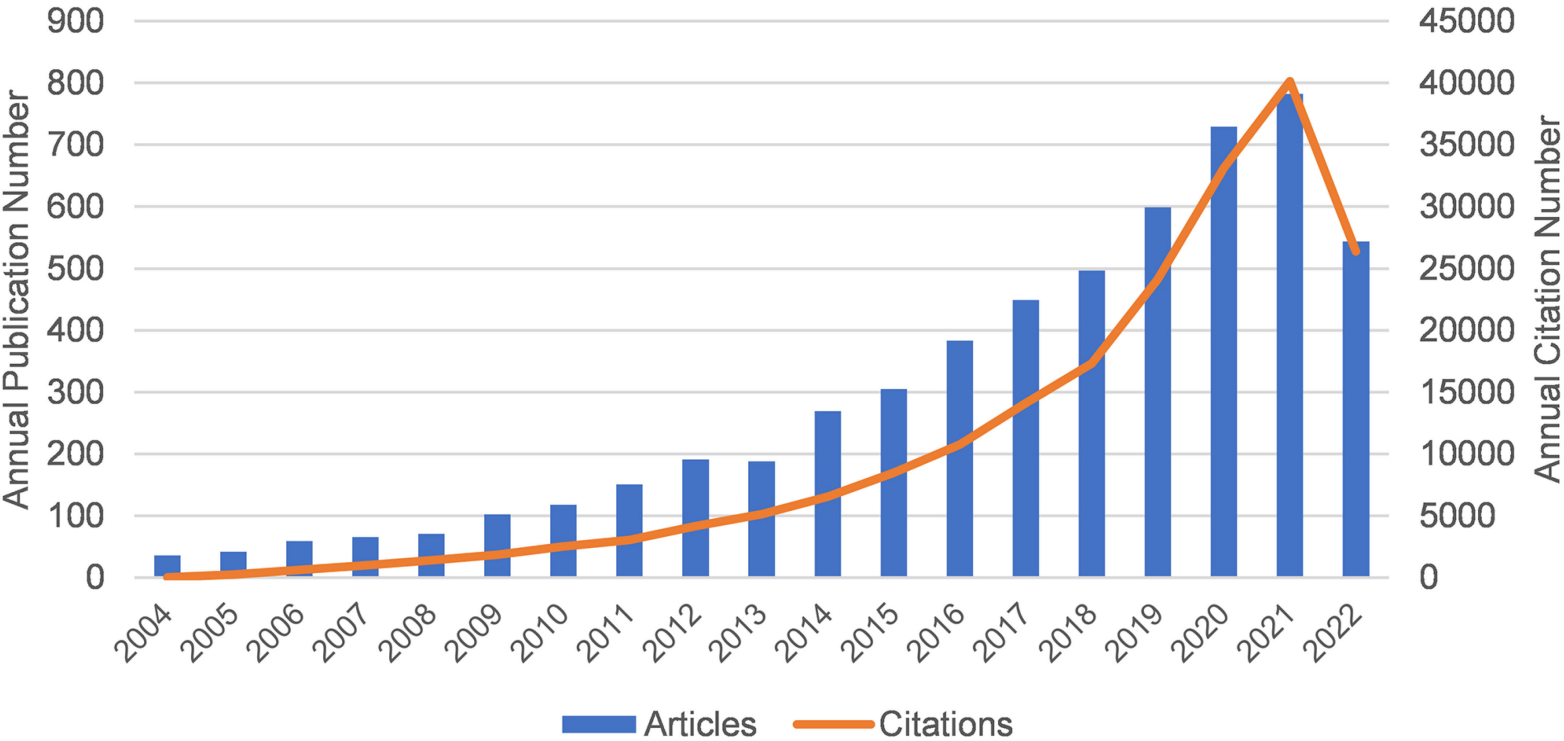
The annual publication volume and annual citation frequency of relevant articles from 2004 to 2022 (2022 data only until September 17).

**Figure 1** shows the annual publication volume and annual citation frequency of relevant articles from 2004 to 2022. Overall, the number of annual publications related to Melanoma biomarkers showed an increasing trend. Among them, the number of publications decreased in 2013, and increased in the rest years. The annual citation frequency related to Melanoma biomarkers showed an increasing trend, and the uptrend of citation frequency increased significantly after 2018. In 2021, the annual publication volume and citation frequency are the highest in history, reaching 782 and 40,121 times.

### 3.2 Distribution of countries or regions

**Figure 2:**
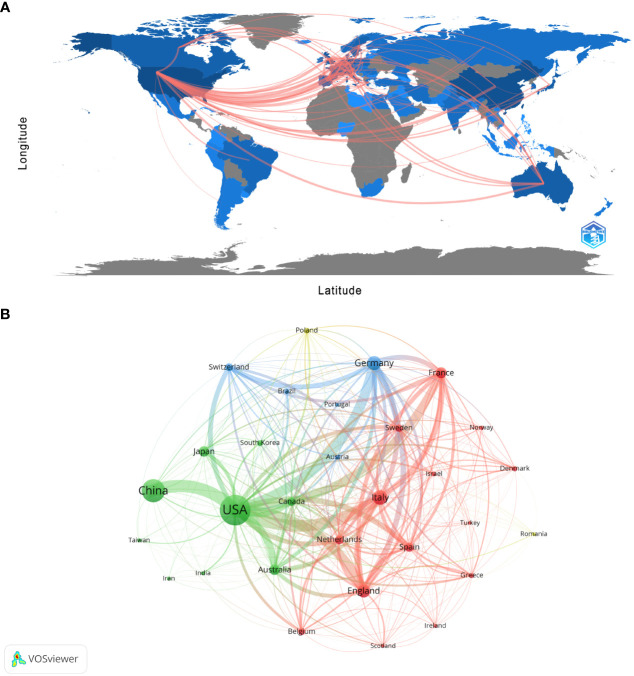
Analysis of melanoma biomarker-related country/region. **(A)** Countries/regions engaged in melanoma-related biomarker research. The connections between countries demonstrate their collaboration and interconnection. **(B)** Visualization of collaboration networks among countries/regions using VOSviewer. The figure illustrates countries/regions with more than 1 document. Nodes of varying colors represent different clusters of countries/regions, and the node size corresponds to their respective prominence.

There are 99 countries/regions participating in the study of Melanoma biomarkers, mainly in the northern hemisphere. It is worth noting that the links between countries/regions are mainly enriched between North America and Europe, North America and East Asia, and there are also strong links between Europe and East Asia, and North America and Oceania (**Figure 2A**). **Table 1** shows the top ten countries/regions in terms of publication volume and citation frequency. The country with the most publications is USA (2039), followed by China (1206) and Italy (521), and the publications of the rest of the countries are less than 500. In terms of citation frequency, the number of USA far surpasses other countries, reaching 120,666 times. In addition, countries such as Italy (25,464), Germany (24,490), China (24,077), France (22,693) and England (20,258) also have high citation frequency. Other countries have less than 20,000 citations. In addition, countries such as South Korea (75.54), France (72.73), and the Netherlands (68.20) have high average citations per article. The United States has an average citation per article of 59.18, ranking 7th among all countries.

**Table 1:** Top 10 countries/regions in terms of number of publications, the frequency of citations and the citations per article.

Rank	Countries/regions	Publications	Countries/regions	Citations	Countries/regions	Citations per article
1	USA	2039	USA	120666	South Korea	75.54
2	China	1206	Italy	25464	France	72.73
3	Italy	521	Germany	24490	Netherlands	68.20
4	Germany	487	China	24077	Sweden	64.24
5	England	401	France	22693	Spain	63.34
6	France	312	England	20258	Romania	59.48
7	Japan	277	Netherlands	14117	USA	59.18
8	Australia	275	Australia	14014	Brazil	53.16
9	Spain	213	Spain	13491	Canada	52.07
10	Netherlands	207	Sweden	9829	Greece	51.47

**Figure 2B** shows the collaboration between the countries involved in the Melanoma biomarker study. In VOSViewer, according to the closeness of cooperation, countries and regions are mainly divided into 3 blocks, which are represented by different colors. The green blocks mainly include countries such as USA, China, Japan, Australia, and Canada, the red blocks mainly include countries such as Italy, France, England, Spain, and the Netherlands, and the blue blocks mainly include countries such as Germany, Switzerland, and Brazil. The thickness of the line between country nodes is related to the strength of connection between countries. The results showed that the connections between USA and countries such as China, Italy, Germany and France were strong, indicating that those countries occupy the core position in the field of Melanoma biomarkers.

### 3.3 Distribution of institutions

Currently, a total of 303 institutions from more than 30 countries have high influence in the field of Melanoma biomarkers. **Table 2** shows the top ten institutions in terms of publication volume and citation frequency. The institution with the most publications is The University of Texas MD Anderson Cancer Center (USA), with 205 publications. Followed by Memorial Sloan Kettering Cancer Center (USA) (116), University of Pittsburgh (USA) (109), Harvard Medical School (USA) (108) and The University of Sydney (Australia) (104), and others are less than 100 articles. The most frequently cited institution is Memorial Sloan Kettering Cancer Center, reaching 14,013 times; followed by The University of Texas MD Anderson Cancer Center (12,256) and Dana-Farber Cancer Institute (USA) (10,212). The remaining institutions have less than 10,000 citations.

**Table 2:** Top 10 institutions in terms of number of articles issued and the frequency of citations.

Rank	Institution	Publications	Institution	Citations
1	The University of Texas MD Anderson Cancer Center (USA)	205	Memorial Sloan Kettering Cancer Center (USA)	14013
2	Memorial Sloan Kettering Cancer Center (USA)	116	The University of Texas MD Anderson Cancer Center (USA)	12256
3	University of Pittsburgh (USA)	109	Dana-Farber Cancer Institute (USA)	10212
4	Harvard Medical School (USA)	108	National Cancer Institute (USA)	8913
5	The University of Sydney (Australia)	104	Massachusetts General Hospital (USA)	8404
6	National Cancer Institute (USA)	85	University of Pennsylvania (USA)	7849
7	Massachusetts General Hospital (USA)	81	Harvard Medical School (USA)	7770
8	Sun Yat-Sen University (China)	78	Yale University (USA)	7324
9	University of Pennsylvania (USA)	76	Harvard University (USA)	7273
10	Dana-Farber Cancer Institute (USA)	71	Stanford University (USA)	5824

**Figure 3:**
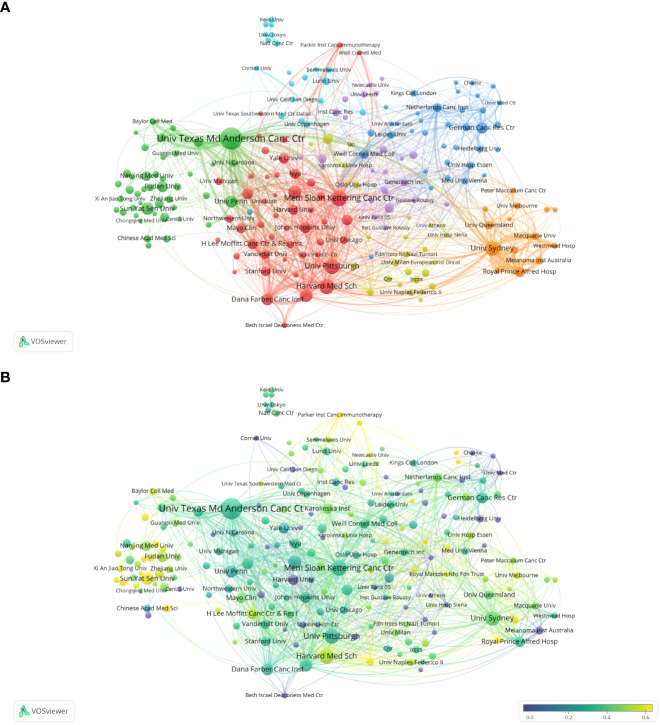
Analysis of melanoma biomarker-related institution. **(A)** Visualization of collaborative network among institutions using VOSviewer. The figure displays institutions with more than 5 documents. Nodes of varying colors represent different clusters of institutions, and the node size corresponds to the frequency of their occurrence. **(B)** Analysis of institution's recent article publication. The heat value of each institution in the past 5 years is calculated by dividing the number of publications in the past 5 years by the total number of publications.

The analysis of research institutions aims to understand the global distribution of Melanoma biomarker- related research and provide opportunities for cooperation. **Figure 3A** shows the collaboration of institutions involved in Melanoma biomarker research. In VOSViewer, according to the closeness of cooperation, the institution is mainly divided into 7 blocks, which are represented by different colors. The red block mainly includes institutions such as Memorial Sloan Kettering Cancer Center, University of Pittsburgh, and Harvard Medical School, the green block mainly includes institutions such as The University of Texas MD Anderson Cancer Center, University of Pennsylvania, and Sun Yat-Sen University, whereas the blue block mainly covers German Cancer Research Center and Netherlands Cancer Institute, the orange block mainly includes institutions such as The University of Sydney and Royal Prince Alfred Hospital, the yellow block mainly contains University of Naples Federico II, and the purple block mainly includes The Institute of Cancer Research and other institutions, the light blue block mainly includes institutions such as Lund University. **Figure 3B** displays publications for each reach institute in the past five years. By dividing the number of Melanoma-related publications of an institution in the past five years by the total number of related publications from 2004 to 2022, the ratio can reflect the contribution of the institution in each five years interval. Nodes with deeper yellow indicate that the institution has a high ratio; nodes with deeper purple indicate that the institution has a low ratio. The results show that the number of papers published by institutions such as Sun Yat-Sen University, Fudan University and Parker Institute for Cancer Immunotherapy has increased significantly in the past five years, indicating that they are emerging institutions in this field. In contrast, Harvard University, Heidelberg University and Melanoma Institute Australia have produced a relatively few related studies in the past five years.

**Figure S1** shows the institutional collaboration network for Melanoma biomarker research. In CiteSpace, each node represents an institution, and the radius of the node increases as its contribution to Melanoma biomarker research increases. The connection between nodes indicates the cooperative relationship between various countries and regions, and the thickness of the link is positively correlated with the depth of cooperation. The betweennness centrality of a node indicates the degree of association between it and other nodes, which is proportional to the size of the purple ring around the surrounding nodes. The results showed that The University of Texas MD Anderson Cancer Center was the most productive institution. In addition, institutions such as Harvard Medical School, University of Sydney and University of Pittsburgh have high productivity. Notably, Karolinska Institute has a high central value, indicating extensive collaboration with other institutions around the world.

### 3.4 Distribution of authors

Co-cited author analysis means that two authors’ documents are cited by a third author at the same time. The higher the co-citation frequency, the closer the academic interest and the research density (35). Through the analysis of the authors with the largest number of publications and co-citation frequency in Melanoma biomarker- related research, it can intuitively reflect the author’s research strength and Melanoma-related research hotspots. A total of 5584 articles published by 22,373 authors were included in this study. **Table 3** shows the top ten authors in terms of publication volume and co-citation frequency. The author with the most publications is Paolo A. Ascierto (Istituto Nazionale Tumori Fondazione G Pascale, Italy) (64), followed by Richard A. Scolyer (Melanoma Institute Australia, Australia) (50) and Georgina V. Long (University of California, USA) (48). The author with the most co-citations is Caroline Robert (Paris-Saclay University, France) (1594), followed by F. Stephen Hodi (Dana-Farber Cancer Institute, USA) (1115) and Suzanne L. Topalian (Johns Hopkins University, USA) (923).

**Table 3:** Top 10 authors in terms of number of publications and the frequency of co-citations.

Rank	Author	Publications	Author	Co-citations
1	Ascierto, Paolo A.	64	Robert, Caroline	1594
2	Scolyer, Richard A.	50	Hodi, F. Stephen	1115
3	Long, Georgina V.	48	Topalian, Suzanne L.	923
4	Schadendorf, Dirk	44	Larkin, James	849
5	Dummer, Reinhard	36	Wolchok, Jedd D.	838
6	Garbe, Claus	36	Ribas, Antoni	757
7	Kirkwood, John M.	33	Long, Georgina V.	706
8	Wolchok, Jedd D.	33	Balch, Charles M.	668
9	Flaherty, Keith T.	32	Weber, Jeffrey S.	574
10	Hoon, Dave S. B.	32	Eggermont, Alexander M. M.	550

**Figure 4:**
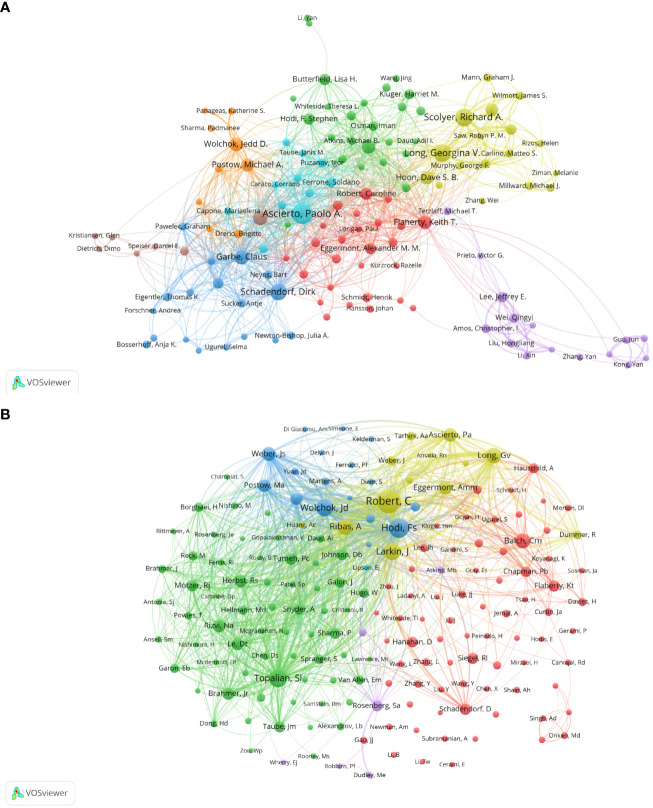
Analysis of melanoma biomarker-related author. **(A)** Visualization of collaborative network among authors using VOSviewer. The figure displays authors with more than 8 documents. Nodes of varying colors represent authors in different clusters, and the node size corresponds to the frequency of their occurrence. **(B)** Visualization of authors' citation networks using VOSviewer. The size of the nodes reflects the frequency of their occurrence.

**Figure 4A** shows the collaboration of the authors involved in the Melanoma biomarker study. In VOSViewer, according to the closeness of cooperation, the author is mainly divided into 7 blocks, which are represented by different colors. The red blocks mainly include authors such as Caroline Robert, Keith T. Flaherty, the blue blocks mainly include Dirk Schadendorf, Claus Garbe, etc., and the green blocks mainly include, F. Stephen Hodi, Lisa H. Butterfield, etc., the yellow block mainly includes authors such as Richard A. Scolyer, Georgina V. Long, the orange block mainly includes authors such as Jedd D. Wolchok, Michael A. Postow, and the purple block mainly includes Qingyi Wei, Jeffery E. Lee and other authors, and the light blue block mainly includes Paolo A. Ascierto, Soldano Ferrone and other authors. The light blue block represented by Paolo A. Ascierto has extensive and close cooperation with other blocks. In contrast, the authors of purple and brown blocks have relatively limited cooperation with authors of other blocks. **Figure 4B** shows the co-citing author relationship network diagram. The results show that the research focus of the authors of Melanoma-related studies is highly homogeneous, mainly divided into 4 blocks. Red blocks include authors such as Charles M. Balch, Dirk Schadendorf, green blocks include authors such as Suzanne L. Topalian, Roy S. Herbst, yellow blocks include authors such as Caroline Robert, James Larkin, and blue Blocks include Jedd D. Wolchok, F. Stephen Hodi and other authors.

### 3.5 Distribution of journals

We used the bibliometric online analysis platform to identify journals with high publication volume and impact in the field of Melanoma biomarkers. The results showed that a total of 532 academic journals had published articles related to Melanoma biomarkers. **Table 4** shows the top ten journals in terms of publication volume and co-citation frequency. The journal with the most publications is Cancers (6.575, Q1) (188), followed by Journal For Immunotherapy Of Cancer (12.487, Q1) (131) and Clinical Cancer Research (13.801, Q1) (126). The journal with the most co-citations is The New England Journal of Medicine (176.079, Q1) (11,688), followed by Journal of Clinical Oncology (50.769, Q1) (11,649) and Clinical Cancer Research (10,521). It is worth noting that the number of publications and co-citations of Clinical Cancer Research ranked third, indicating that it has a strong influence in Melanoma-related fields.

**Table 4:** Top 10 journals in terms of number of publications, the frequency of co-citations, and the corresponding IF (JCR 2021) and JCR quartile.

Rank	Journals	Publications	Journals	Co-citations
1	Cancers 6.575(Q1)	188	The New England Journal of Medicine 176.079(Q1)	11688
2	Journal For Immunotherapy Of Cancer 12.487(Q1)	131	Journal of Clinical Oncology 50.769(Q1)	11649
3	Clinical Cancer Research 13.801(Q1)	126	Clinical Cancer Research 13.801(Q1)	10521
4	Plos One 3.752(Q2)	113	Cancer Research 13.312(Q1)	9349
5	Frontiers In Oncology 5.738(Q2)	107	Nature 69.504(Q1)	7636
6	Melanoma Research 3.199(Q2)	97	Proceedings of the National Academy of Sciences of The United States Of America 12.779(Q1)	5335
7	Oncotarget -	90	Science 63.798(Q1)	5334
8	Frontiers In Immunology 8.786(Q1)	81	Cell 66.850(Q1)	4761
9	International Journal Of Molecular Sciences 6.208(Q1)	76	Plos One 3.752(Q2)	4535
10	Journal Of Translational Medicine 8.459 (Q1)	75	The LancetOncology 54.433(Q1)	3702

**Figure 5:**
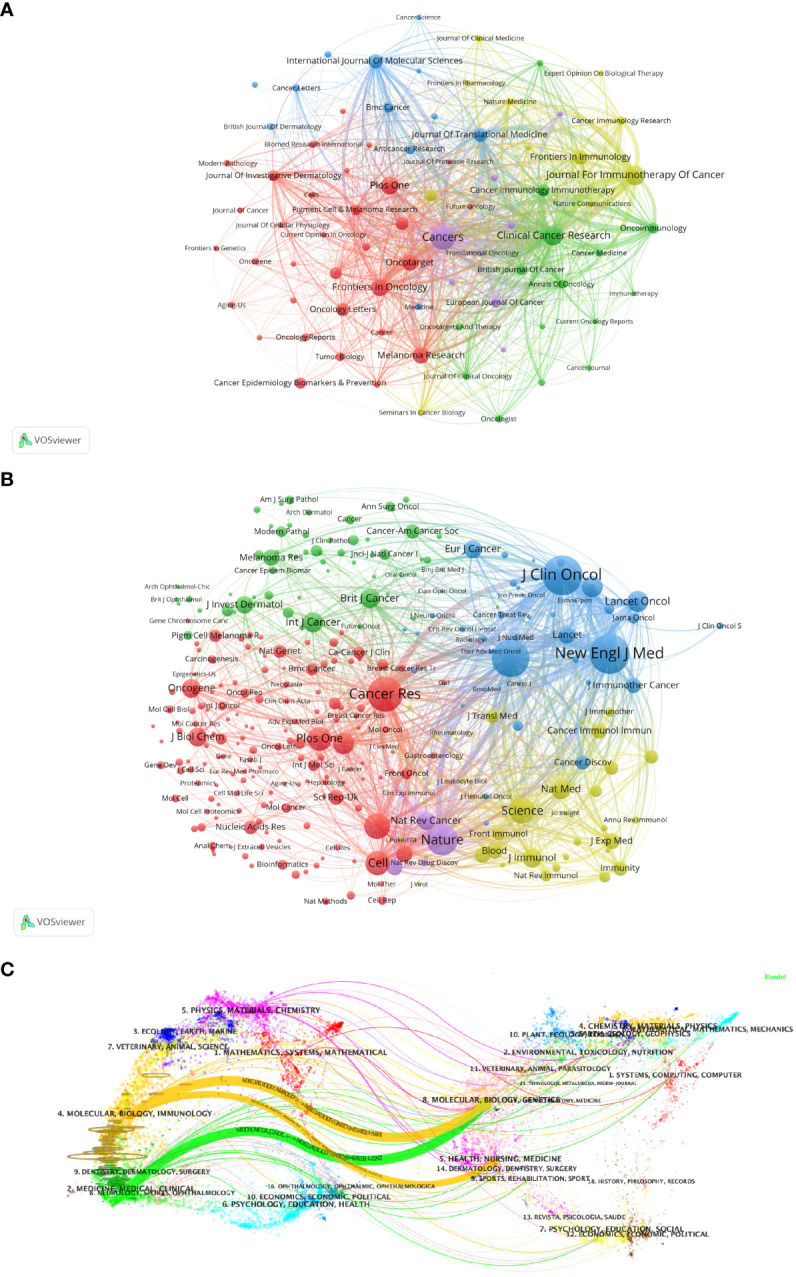
Analysis of melanoma biomarker-related journal. **(A)** Visualization of collaborative network among journals using VOSviewer. The figure displays journals with more than 10 documents. Nodes of varying colors represent journals in different clusters, and the node size corresponds to the frequency of their occurrence. **(B)** Visualization of journals' citation networks using VOSviewer. The size of the nodes reflects the frequency of their occurrence. **(C)** Dual-map overlay of journals, with citing journals on the left and cited journals on the right. Colored paths indicate citation relationships.

**Figure 5A** visualizes the journals that published articles related to Melanoma biomarkers and the relationship between them. In VOSviewer, journals are mainly divided into 5 blocks of different colors according to the similarity of the them. The red block mainly includes journals such as Frontiers In Oncology, Plos One, Malanoma Research, and Journal Of Investigative Dermatology, the green block mainly includes journals such as Clinical Cancer Research, Oncoimmunology, British Journal of Cancer, and the blue block mainly includes International Journal Of Molecular Sciences, Journal Of Translational Medicine, BMC Cancer and other journals, the yellow block mainly includes journals such as Journal For Immunotherapy Of Cancer, Frontiers In Immunology, Cancer Immunology Research, and the purple block mainly includes journals such as Cancers and European Journal Of Cancer. The research fields of the journals in the red block are mainly concentrated in the field of oncology (Frontiers In Oncology, Oncotarget, Melanoma Research, etc.); the research fields of the journals in the yellow block are mainly concentrated in the field of immunology (Frontiers In Immunology, Cancer Immunology Research, Journal For Immunotherapy Of Cancer, etc.); the research fields of green block journals are to a certain extent manifested in the intersection of oncology and immunology (Cancer Immunology Immunology Immunotherapy, Oncoimmunology, etc.), oncology and clinical Crossover (Clinical Cancer Research, Journal Of Clinical Oncology, etc.); similar to the red block, the journals in the purple block are also mainly focused on oncology (Cancers, European Journal Of Cancer, etc.); while the journals in the blue block covers relatively broad research fields with no obviously focused research field. According to the co-citation frequency, Melanoma biomarker- related journals are mainly divided into 4 blocks with similar research directions (**Figure 5B**). The red blocks mainly focus on BIOCHEMISTRY & MOLECULAR BIOLOGY (JOURNAL OF BIOLOGICAL CHEMISTRY, Cell, Oncogene, etc.), the green blocks mainly focus on the field of oncology (BRITISH JOURNAL OF CANCER, INTERNATIONAL JOURNAL OF CANCER, etc.), and the blue blocks Mainly focus on the field of clinical and oncology (NEW ENGLAND JOURNAL OF MEDICINE, LANCET ONCOLOGY, JOURNAL OF CLINICAL ONCOLOGY, etc.), and the yellow block mainly focuses on the direction of immunology (Frontiers In Immunology, Journal Of Immunology, etc.).

We used knowledge flow analysis to explore the evolution process of knowledge citation and co-citation between citing journals and cited journals (36). The dual-map overlay of journals shows the distribution of topics, citation trajectories, and movement of research centers across academic journals (**Figure 5C**) (36, 37). The label on the left of the Dual-map represents citing journals, and the label on the right represents cited journals. The citation connection colored curve originating from the citing map and pointing to the cited map shows the complete context of the citation. In the citing map, the more papers a journal publishes, the longer the vertical axis of the ellipse; the larger the number of authors, the longer the horizontal axis of the ellipse. The topics of Citing Journals are mainly MOLECULAR, BIOLOGY, IMMUNOLOGY, MEDICINE, MEDICAL, CLINICAL, namely research frontier. The topics of Cited Journals are mainly MOLECULAR, BIOLOGY, GENETICS, HEALTH, NURSING, MEDICINE, DERMATOLOGY, DENTISTRY, SURGERY, namely the knowledge base.

### 3.6 Keyword analysis

**Figure 6:**
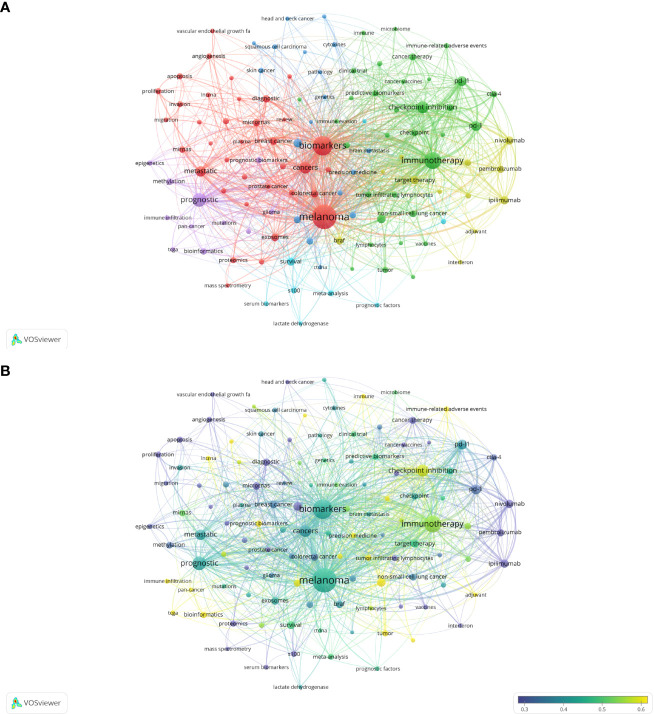
Analysis of melanoma biomarker-related keyword. **(A)** Visualization of keyword collaboration network using VOSviewer. The figure displays keywords that occur more than 15 times. Nodes of varying colors represent different clusters of keywords, and the node size corresponds to their frequency. **(B)** Analysis of keyword's recent article publication. The heat value of each keyword in the past 5 years is calculated by dividing the number of publications in the past 5 years by the total number of publications.

As the core overview of the content of the article, keywords can be used to analyze the frontiers of Melanoma biomarker research. **Table 5** shows the top 20 keywords with the frequency of occurrence. The most frequently occurring keyword was “melanoma” (1781), followed by “biomarkers” (1026) and “immunotherapy” (677). “prognostic” (417), “checkpoint inhibition” (294) and “cancers” (278) were also frequently occurring keywords, indicating that they are hot topics in the field of Melanoma biomarkers. The occurrence frequency of other keywords is less than 200 times. **Figure 6A** shows the keyword co-occurrence network diagram. Keywords with close co-occurrence relationship are clustered into one category, mainly with 4 larger blocks, which are represented by different colors. The keywords in the red block are mainly related to melanoma diagnostic biomarkers (diagnostic, metastatic, migration), and the keywords in the green block are mainly related to melanoma treatment-related biomarkers (immunotherapy, cancer therapy, pd-l1, c checkpoint inhibition), the keywords in the purple block are mainly related to melanoma prognosis-related biomarkers (prognostic, prognostic biomarkers), the keywords in the yellow block are mainly related to PD-L1 inhibitors (nivolumab, pembrolizumab, ipilimumab). It is worth noting that the red blocks have very extensive connections with other blocks, indicating their cross-fields in various related research fields. **Figure 6B** shows the popularity analysis of keywords in the past 3 years. By dividing the frequency of occurrence of keywords in the past 3 years by the total frequency of occurrence, we obtained the popularity value of the keyword in the past 3 years. The yellowish color of the node means that its popularity has been high in recent years, and the purple color of the node means that its popularity has been low in recent years. The results show that keywords such as c checkpoint inhibition, immune, bioinformatics have become more popular in the past three years. On the contrary, diagnostic, micrornas, epigenetics, apoptosis, s100, pd-1, etc. have become relatively less popular in the past three years.

**Table 5:** Top 20 keywords in terms of frequency of occurrence and the corresponding total link strength.

Rank	Keyword	Occurrences	Total link strength
1	melanoma	1781	3421
2	biomarkers	1026	2465
3	immunotherapy	677	1848
4	prognostic	417	925
5	checkpoint inhibition	294	787
6	cancers	278	565
7	metastatic	198	470
8	pd-l1	180	585
9	pd-1	172	613
10	immunohistochemistry	130	217
11	tumor microenvironment	125	340
12	nivolumab	119	418
13	target therapy	112	344
14	ipilimumab	110	388
15	survival	107	246
16	breast cancer	94	212
17	exosomes	92	220
18	colorectal cancer	89	173
19	diagnostic	86	215
20	micrornas	85	218

**Figure 7:**
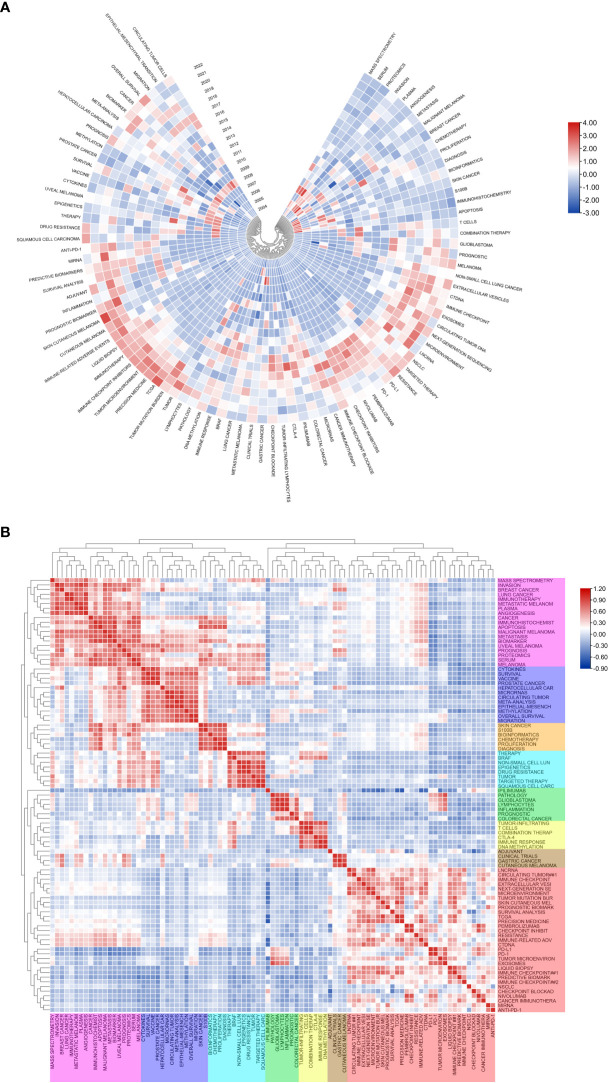
Heatmap analysis of melanoma biomarker-related keywords. **(A)** Annual heatmap from 2004 to 2022. The annual heat value of each keyword is obtained by dividing the number of citations in that year by the total number of citations in that year. **(B)** Keyword relevance heatmap. Keywords with high popularity in similar time periods are clustered into one category and marked with different colors.

**Figure 7A** shows the annual popularity of keywords from 2004 to 2022 (the number of citations of a keyword in a year/total citations of queried keywords in a year). In recent years, keywords such as tumor-infiltrating lymphocytes and breast cancer have had relatively low annual popularity. In contrast, skin cutaneous melanoma, TCGA (The Cancer Genome Atlas Program), immunotherapy, tumor microenvironment and immune-related adverse events have relatively high annual prevalence, proving that these keywords represent emerging frontier areas that may become the hotspot of future melanoma biomarker research. **Figure 7B** shows the correlation between popular keywords from 2004 to 2022, where keywords with high popularity in similar periods are clustered into different clusters marked with different colors. The results suggest that the pathogenesis of melanoma is closely related to the immune defense status of the body. For example, tumor and epigenetics, targeted therapy and drug resistance are closely correlated. Numerous studies have shown that abnormal epigenetic modifications lead to tumorigenesis, and epigenetic detection of tumor-related mutations allows for early and more accurate diagnosis and more precise treatment of tumors (38, 39). In addition, drug resistance has been a challenge for clinical treatment, and targeted therapy can help overcome drug resistance (40, 41). Immune checkpoints usually appear together with keywords for tumor-related diseases such as melanoma, NSCLC (non-small cell lung cancer), and breast cancer, suggesting that immune checkpoints are closely related to the prognosis of tumor-related diseases and can improve effective strategies for clinical treatment (42, 43).

### 3.7 Highly cited reference analysis

**Table 6** shows the top 15 articles with citation frequency and annual average citation frequency. The article with the highest citation frequency is “Predictive correlates of response to the anti-PD-L1 antibody MPDL3280A in cancer patients” (RS Herbst et al., 2014) (3399), in which Herbst et al. developed a high-affinity human monoclonal immunoglobulin G1 antibody (MPDL3280A) that specifically binds PD-L1 for clinical use to block PD-L1 and its ligands PD-1 or B7.1 (CD80), so as to achieve the purpose of enhancing the anti-cancer immunity of patients. Among them, the positive response rate to PD-L1 inhibition in melanoma patients reached 26% (11 of 43) (44). “Detection of Circulating Tumor DNA in Early- and Late-Stage Human Malignancies” (C Bettegowda et al., 2014) (2675) is the second most cited article. Bettegowda et al. evaluated the ability of circulating tumor DNA (ctDNA) to detect different types of tumors based on digital polymerase chain reaction technology, and the results showed that melanoma patients had sufficient levels of ctDNA to detect (45). In terms of average annual citation frequency, “Predictive correlates of response to the anti-PD-L1 antibody MPDL3280A in cancer patients” is still ranked first, reaching 377.67 times per year; followed by “Atezolizumab in patients with locally advanced and metastatic urothelial carcinoma who have progressed following treatment with platinum-based chemotherapy: a single-arm, multicentre, phase 2 trial” (Jonathan E. Rosenberg et al., 2016) (336.71 per year), which mainly evaluates the engineered human immune system that can selectively bind PD-L1 Efficacy of atezolizimab, a globulin G1 monoclonal antibody, in patients with metastatic urothelial carcinoma (46).

**Table 6:** Top 15 articles in terms of frequency of citation.

Rank	Article Title	Source Title	Authors	Year	Cited	DOI
1	Predictive correlates of response to the anti-PD-L1 antibody MPDL3280A in cancer patients	Nature	Herbst, Roy S. et al.	2014	3399	10.1038/nature14011
2	Detection of Circulating Tumor DNA in Early- and Late-Stage Human Malignancies	Science Translational Medicine	Bettegowda, Chetan et al.	2014	2675	10.1126/scitranslmed.3007094
3	Atezolizumab in patients with locally advanced and metastatic urothelial carcinoma who have progressed following treatment with platinum-based chemotherapy: a single-arm, multicentre, phase 2 trial	Lancet	Rosenberg, Jonathan E. et al.	2016	2357	10.1016/S0140-6736(16)00561-4
4	Cancer immunotherapy: moving beyond current vaccines	Nature Medicine	Rosenberg, Steven A. et al.	2004	2247	10.1038/nm1100
5	Systematic identification of genomic markers of drug sensitivity in cancer cells	Nature	Garnett, Mathew J. et al.	2012	1574	10.1038/nature11005
6	Nivolumab plus Ipilimumab in Lung Cancer with a High Tumor Mutational Burden	The New England Journal of Medicine	Hellmann, M. D. et al.	2018	1534	10.1056/NEJMoa1801946
7	Mechanism-driven biomarkers to guide immune checkpoint blockade in cancer therapy	Nature Reviews Cancer	Topalian, Suzanne L. et al.	2016	1433	10.1038/nrc.2016.36
8	PD-L1 (B7-H1) and PD-1 pathway blockade for cancer therapy: Mechanisms, response biomarkers, and combinations	Science Translational Medicine	Zou, Weiping et al.	2016	1341	10.1126/scitranslmed.aad7118
9	PD-L1 Expression as a Predictive Biomarker in Cancer Immunotherapy	Molecular Cancer Therapeutics	Patel, Sandip Pravin et al.	2015	1268	10.1158/1535-7163.MCT-14-0983
10	Exosomal PD-L1 contributes to immunosuppression and is associated with anti-PD-1 response	Nature	Chen, Gang et al.	2018	1180	10.1038/s41586-018-0392-8
11	Tumor Mutational Burden as an Independent Predictor of Response to Immunotherapy in Diverse Cancers	Molecular Cancer Therapeutics	Goodman, Aaron M. et al.	2017	1159	10.1158/1535-7163.MCT-17-0386
12	The evolving landscape of biomarkers for checkpoint inhibitor immunotherapy	Nature Reviews Cancer	Havel, Jonathan J. et al.	2019	1017	10.1038/s41568-019-0116-x
13	Signatures of T cell dysfunction and exclusion predict cancer immunotherapy response	Nature Medicine	Jiang, Peng et al.	2018	1007	10.1038/s41591-018-0136-1
14	Comprehensive analyses of tumor immunity: implications for cancer immunotherapy	Genome Biology	Li, Bo et al.	2016	983	10.1186/s13059-016-1028-7
15	Full-length mRNA-Seq from single-cell levels of RNA and individual circulating tumor cells	Nature Biotechnology	Ramskold, Daniel et al.	2012	941	10.1038/nbt.2282

**Figure 8:**
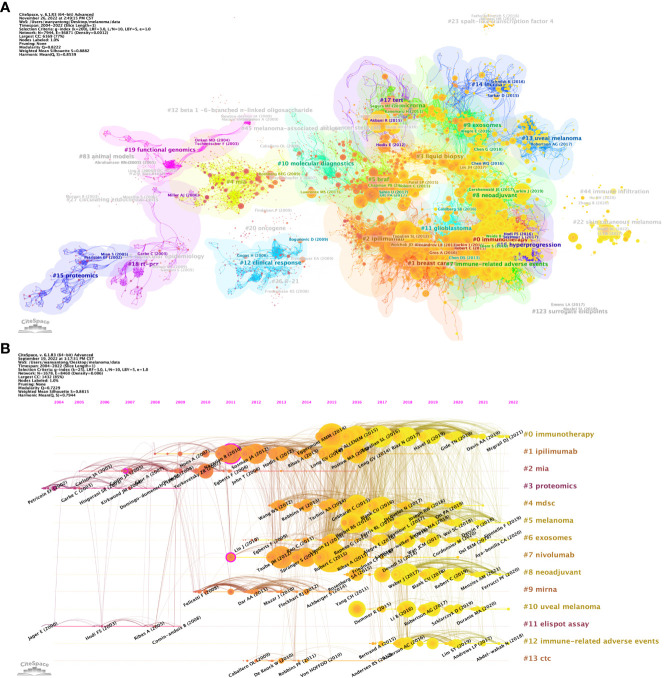
Analysis of melanoma biomarker-related reference. **(A)** Analysis of reference network using CiteSpace. The size of each node represents the frequency of co-citation for the corresponding article. **(B)** Timeline view of reference. A horizontal line represents a cluster, with smaller numbers indicating larger clusters (#0 represents the largest cluster). Node size represents co-citation frequency, and the links between nodes indicate co-citation relationships. The occurrence year of each node indicates the initial co-citation time.

Article co-citation analysis analyzes the relationship between articles by analyzing the co-citation frequency of articles. **Figure 8A** shows the relationship between the studies. The authors and years of the documents whose co-citation frequency has exploded are marked in the figure, and the documents are clustered according to the closeness of the association. The results showed that, from 2002 to 2009, the research in the field of melanoma mainly had two independent development paths. In one of the paths, relevant early research mainly focused on 5 closely related clusters, including #27 (circulating endothelial cells), #25 (molecular epidemiology), #83 (aninal mode), #29 (survivin), #19 (functional genomics) and #18 (rt-pcr), these clusters subsequently developed into 2 clusters of #4 (mia) and #20 (oncogene). In another path, the earliest cluster was #15 (proteomics), which later developed into two clusters, #26 (IL-21) and #12 (clinical response). These two developmental paths eventually converged into a single cluster, #10 (molecular diagnostics). After 2010, cluster #10 developed into clusters #5 (braf), #2 (iphmmumab), #1 (breast cancer) and #0 (immunotherapy). It is noteworthy that within these clusters, the number of outbreak documents was significantly increased and their linkages between each other were significantly enhanced. Subsequently, Melanoma-related research developed into four relatively independent directions at different time points, namely #14 (lncrna) appeared around 2015, #13 (uveal melanoma) emerged around 2017, and #44 appeared around 2020 (immune infiltration) and #22 (skin cutaneous melanoma) appeared around 2022.

In CiteSpace, a timeline graph shows articles with high co-citations in each cluster over time (**Figure 8B**). #0 (immunotherapy) was the largest cluster, followed by #1 (ipilimumab), #2 (mia) and #3 (proteomics). In the field of immunotherapy, Eggermont AMM (2014), VAN ALLENEM (2015), etc. are earlier high-impact studies, while Mcgrail DJ (2021) is one of the latest high-impact studies. From the timeline, #11 (elispot assay) and #3 (proteomics) are the first two clusters, and #10 (uveal melanoma) is the latest one. Notably, the largest cluster #0 (immunotherapy) is also a late cluster, indicating that it is a hot topic that has emerged in recent years. Among the 13 clusters, research related to 6 clusters is still ongoing, indicating that these research directions are still hot spots in Melanoma-related research.

**Figure 9:**
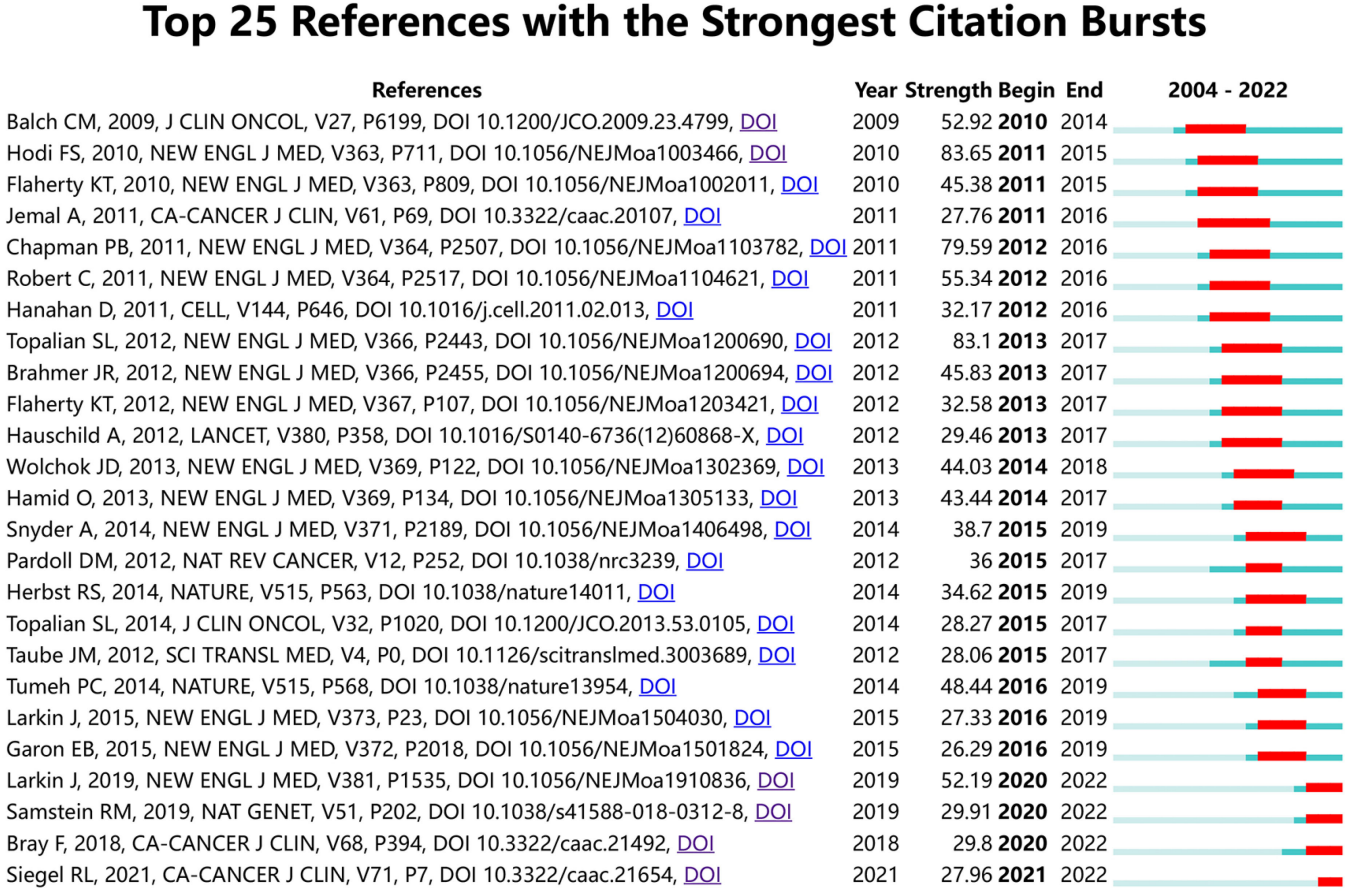
The top 25 references with the strongest citation bursts.

**Figure 9** shows the top 25 references with the strongest citation bursts. The earliest burst of citations occurred in 2010, and the title of this article is “Final Version of 2009 AJCC Melanoma Staging and Classification”, which was published in Journal of Clinical Oncology by Charles M. Balch et al. in 2009 (47). The article titled “Improved Survival with Ipilimumab in Patients with Metastatic Melanoma” published in The New England Journal of Medicine by F. Stephen Hodi et al. in 2010 has the highest burst strength (Strength = 83.65) (48). 2015 was the year with the most citation outbreaks, with a total of 5 citation outbreaks, and the outbreak lasted until 2019; followed by 2013, with 4 citations. In addition, articles such as “ Five-Year Survival with Combined Nivolumab and Ipilimumab in Advanced Melanoma “ by James Larkin, F.R.C.P. et al. and “Tumor mutational load predicts survival after immunotherapy across multiple cancer types” by Robert M. Samstein et al. are recent citing outbreak articles, and their outbreaks continue (49, 50).

## 4 Discussion

### 4.1 General information

The analysis of this study is based on 5584 articles related to melanoma biomarkers from 99 countries and 22,373 authors in the WoSSC database from January 1, 2004 to September 17, 2022. Overall, the number of articles and citations are increasing year by year, indicating that the field is attracting more and more attention. Compared with 2011, the frequency of citations and the number of publications in 2021 have increased by about 5 times and 13 times, respectively. It is worth noting that the number of studies in this field increased significantly in 2018, which may attribute to the fact that James Allison and Tasuku Honjo won the Nobel Prize in Physiology or Medicine for their outstanding contributions to the study of CTLA-4 and PD-1, increasing the popularity of the field.

The country/region analysis shows that the United States has far more publications and citation frequency than other countries, and is the most influential country in the field of melanoma biomarkers (**Table 1**). Most of the top institutions in this field are from the United States, and 8 of the top ten institutions in terms of publication volume are from the United States; all the top ten institutions in the citation frequency are also from the United States (**Table 2**). Among them, The University of Texas MD Anderson Cancer Center has the most publications and the second most citations, while Memorial Sloan Kettering Cancer Center has the most citations and the second most publications, indicating that these two institutions from the United States have the most important influence in this field. In terms of cooperation, the United States has the strongest and most links with other countries, indicating that it is a research center in this field (**Figure 2**). In addition to the United States, Italy, China, Germany, France and other countries with a high number of publications and citation frequency have strong influence in this field, and have extensive exchanges and cooperation with other countries.

The author analysis shows that Caroline Robert from Paris-Saclay University is the author with the most co-citations, indicating that he has outstanding influence in the field of melanoma markers. The second most cited author is F. Stephen Hodi from Dana- Farber Cancer Institute. It is worth noting that the article titled “Predictive correlates of response to the anti-PD-L1 antibody MPDL3280A in cancer patients” published by Hodi et al. on Nature in 2014 not only has the most citations in this field, but also has the most annual average citations. Suzanne L. Topalian has the third most co-citations. “Mechanism-driven biomarkers to guide immune checkpoint blockade in cancer therapy” published by Topalian et al. in 2016 ranks in the top ten in terms of citations and annual average citations, and has a high influence in this field (51). In addition, Georgina V. Long and Jedd D. Wolchok among the top ten authors in the number of publications are also the top ten authors in the number of co-citations, which shows that they are also authoritative figures in this field (**Tables 3**, **6**).

Analysis of journals shows that Cancers, Journal For Immunotherapy Of Cancer, which ranks the top in the number of publications, and The New England Journal of Medicine, Journal of Clinical Oncology, and Clinical Cancer Research, which ranks the top in co-citation frequency, have strong competitiveness in this field. Influence. 5 of the top ten papers in terms of citation frequency and 6 of the top 10 papers in annual average citation frequency are from Nature and its sub-journals such as Nature Review Cancer, Nature Medicine, etc. top journals (**Tables 4**, **6**). It is worth noting that among the top ten journals with co-citation frequency, The New England Journal of Medicine is a journal in the field of MEDICINE, Cell is a journal in the field of BIOCHEMISTRY & MOLECULAR BIOLOGY, Clinical Cancer Research, Journal of Clinical Oncology and Cancer Research belong to the field of ONCOLOGY, which is consistent with the dual-map analysis results in **Figure 5C**.

### 4.2 Hot topics and frontiers

Keyword analysis is helpful to understand the frontiers and hotspots of melanoma molecular markers. In existing studies, high-frequency keywords include “Melanoma”, “ Biomarkers”, “Immunotherapy”, “Prognostic”, “Checkpoint Inhibition”, “PD-L1”, “Immunohistochemistry”, etc. (**Table 5**), which is closely related to the diagnosis, treatment and prognosis of melanoma. The results of **Figure 5A** confirm this conclusion. In the keyword co-occurrence network, keywords are summarized into several main directions, including biomarkers in the diagnosis, treatment, and prognosis of melanoma. The high expression of some biomarkers in melanoma makes them suitable as a diagnostic marker for early screening of melanoma (52). In the red block in the keyword co-occurrence graph, “micrornas” and “angiogenesis” are commonly used biomarkers for the diagnosis and evaluation of melanoma, while “mass spectrometry” and “proteomics” are commonly used detection techniques. On the other hand, in the melanoma treatment keywords block, the more prominent keywords are “checkpoint inhibition”, “immunotherapy”, “PD-1” and “PD-L1”. In recent years, immunotherapy against melanoma has shifted from cytokine-based therapy to antibody-mediated immune checkpoint inhibition, including programmed cell-death protein 1 (PD-1) (53). Previous studies have shown that tumor cells can escape immune surveillance by upregulating PD-1, and anti- PD-1 therapy can play a role in melanoma patients (1, 54). It is worth noting that in the keyword co-occurrence network diagram, PD-L1 inhibitors “ipilimumab”, “nivolumab”, and “pembrolizumab” also appear as a separate cluster (yellow). In addition, among the top 10 most cited documents, 6 of them are related to PD-L1 inhibition. These results all indicate that studies related to PD-1/PD-L1 inhibition are highly popular in this field. In addition, the keyword “CTLA-4” is also a relatively popular checkpoint inhibition, which plays an important role in the immunotherapy of melanoma. In addition, some melanoma biomarkers can reflect the degree of tumor expansion and deterioration during treatment, and have good prognostic evaluation value (55). In the purple block, indicators such as “methylation” and “epigenetics” have been proven to be used as the evaluation of melanoma prognosis. **Figure 7** shows that the heat of the field of melanoma treatment-related biomarkers continues to increase, mainly including keywords such as prognostic biomarker, immunotherapy and immune-related adverse events. In addition, the popularity of multi-omics continues to increase, including keywords such as TCGA, tumor mutation burden and next-generation sequencing. These findings underline the growing significance of these research domains and highlight their substantial impact on the field of biomarkers in melanoma.

#### 4.2.1 Melanoma diagnostic biomarker

Melanoma diagnostic biomarkers can be divided into five categories, including visual features, histopathology, morphology, immunohistochemistry, and serological molecular biomarkers (56). In clinical practice, the visual distinction between benign nevus and malignant melanoma mainly follows the “ABCDE” principle, that is, asymmetry, border irregularity, color change, diameter (>6 mm), and degree of evolution (57). Histopathologically, ulceration, mitotic rate, lymphovascular invasion, and neural invasion are common features of melanoma (58). Optical Coherence Tomography (OCT), Reflectance Confocal Microscopy (RCT), High-frequency ultrasound (HFUS) and other techniques can be used to examine the morphological features of the lesion to assist in the diagnosis of melanoma (56). Common morphological features include disorganized lesion tissue architecture, the presence of atypical melanocytes, atypical keratinocytes in the superficial skin, and hypervascular lesions (59–61). With the gradual elucidation of the molecular mechanism of melanoma pathogenesis, relying on immunohistochemistry (IHC), more and more researchers and clinicians use molecular biomarkers to assist the diagnosis of melanoma (62). Melan-A is a melanocyte differentiation antigen expressed in melanocytes, melanoma and retinal pigment epithelial cells (16). There is evidence that Melan-A has high sensitivity and specificity in differentiating melanoma from non-melanocytic tumors (63). HMB-45 is a 100 kD glycoprotein, and studies have shown that it has higher specificity than Melan-A in the diagnosis of melanoma patients (64). Tyrosinase, an enzyme involved in melanogenesis in melanosomes, is also highly sensitive to primary melanoma (10). Chondroitin sulfate proteoglycan 4 (CSPG4) is another membrane-bound proteoglycan used in the diagnosis of melanoma, and its sensitivity to melanoma under immunostaining is greater than 85% (16, 65). In addition, S100 protein family, Microphthalmia-associated transcription factor (MITF), SOX10, etc. have been proven to be diagnostic molecular markers for melanoma (18, 66, 67). On the other hand, melanoma serological markers such as lactate dehydrogenase (LDH) have been proven to have diagnostic evaluation value in many studies (68). In addition, epigenetic factors such as abnormal DNA methylation and miRNA are closely related to the development of melanoma, which has potential significance in the diagnosis of melanoma (69, 70).

#### 4.2.2 Predictive biomarkers for melanoma treatment response

Biomarkers can identify melanoma before it becomes overtly symptomatic and significantly improve melanoma treatment outcomes (52). Accompanying with our understanding of the mechanisms by which cancer cells evade the immune system, new cancer therapies such as cancer vaccines, adoptive cell therapy, and immunomodulatory approaches continue to emerge (71–74). For metastatic melanoma, the most effective treatment at present is the application of immune checkpoint inhibitors, mainly including PD-1/PD-L1 and CTLA4 antibodies (75, 76). Similar to the effect of PD-1, CDLA4 expressed in T-reg cells recognizes B7-1/2 receptors on APCs (Antigen-presenting cells) and competes with CD28 on T cells for binding to B7-1/2 receptors, thereby suppressing the immune response. By blocking its binding to the corresponding ligand, the suppressed immune response in cancer patients can be stimulated (3). Currently approved c checkpoint inhibitors for the treatment of melanoma include an anti-CTLA-4 antibody ipilimumab, an anti-PD-1 antibodies nivolumab and pembrolizumab, correspondingly, their related research has also proved to be one of the hot topics in our result (77–79). With the rapid development of melanoma therapy, the potential role of biomarkers in the prediction of response has received extensive attention. For example, PD-L1 immunohistochemistry has been used in the prediction of treatment response, considering that PD-L1 expression is significantly associated with response rate, progression-free survival, and overall survival in melanoma (7). In addition, monitoring of BRAF mutation status is critical in determining whether a patient may benefit from BRAF inhibitor therapy (80). In mucosal melanoma, there is evidence that activating mutations in c-kit may predict patient sensitivity to the kinase inhibitor imatinib (81).

#### 4.2.3 Melanoma prognostic biomarker

Robust and reliable melanoma biomarkers help to assess patients’ prognostic risk and select more beneficial treatment options. In current clinical practice, Breslow thickness, mitotic rate, and ulceration are the most important prognostic markers in the histopathological criteria of melanoma (13). In addition, the status of sentinel lymph node (SLN) is also an important prognostic indicator in melanoma (82). In recent years, the number of studies on immunohistochemical biomarkers predicting melanoma prognosis has grown rapidly (19, 83). Proliferation markers can reflect the number of cells in the cell cycle in the lesion, and are one of the indicators for evaluating the malignancy of melanoma (84). As a nuclear antigen highly expressed during the active phases of the cell cycle (G1, S, G2, and M), Ki-67 has been shown to be closely associated with melanoma prognosis (85). In thicker melanomas (>1mm), Ki-67 may be a better prognostic predictor than mitotic rate (20). Phosphohistone H3 (PHH3) is also a common mitotic marker, and studies have shown that it can be used as a more precise prognostic marker than Ki-67, but more evidence is still needed to support this conclusion (3). Melanoma cell adhesion molecules (MCAMs) are commonly expressed on endothelial and smooth muscle cells in adult tissues (86). Although MCAM is less expressed in malignant tumors and benign nevi, it can be highly specifically expressed in melanoma cells and has been shown to be an independent predictor of melanoma prognosis (52, 87, 88). Metallothioneins are low-molecular-weight proteins that bind heavy metals, and their overexpression has been shown to be independently associated with tumor development and metastasis (89, 90). In addition to playing a role in diagnosis, serological markers such as LDH, S100, and C-reactive protein (CRP) also play an important role in the evaluation of melanoma prognosis (91–93).

## 5 Conclusion

In this study, we used bibliometric analysis to review the trends, hotspots, and frontiers of melanoma biomarker- related research in the past two decades. The number and citation frequency of melanoma biomarker- related studies are generally increasing year by year, and the importance of this field has been recognized globally. The United States is the core country for research on melanoma biomarkers and has important influence in this field accompanied by extensive cooperation with other countries. The New England Journal of Medicine, Clinical Cancer Research, etc. are highly influential journals in this field, and Robert, Caroline, Hodi, F. Stephen, etc. are authoritative authors in this field. Melanoma diagnosis, treatment and prognosis-related biomarkers are hot topics in this field. The application of melanoma biomarkers in the prediction of immunotherapy effect may be a key direction of future research. These findings provide researchers and policy makers with a comprehensive perspective to fully understand the field of melanoma molecular marker research.

## Data availability statement

Publicly available datasets were analyzed in this study. This data can be found here: https://clarivate.com/products/scientific-and-academic-research/research-discovery-and-workflow-solutions/web-of-science/web-of-science-core-collection/.

## Author contributions

JC, TS, and JL: study conception and design. YW, JS, and YH: study conduct. YW: data analysis. YW, JS, and YH: full access to all the data in the study, take responsibility for the integrity of the data and the accuracy of the data analysis, data interpretation, and drafting of the manuscript. YW, JS, YH, LL, TS, and JL: critical revision of the manuscript for important intellectual content. All authors contributed to the article and approved the submitted version.
