# Supplementary material for: Mapping knowledge landscapes and emerging trends of the biomarkers in melanoma: a bibliometric analysis from 2004 to 2022
Source: Front Oncol. 2023 Jun 23;13:1181164. doi: 10.3389/fonc.2023.1181164 (PMC10327294; doi:10.3389/fonc.2023.1181164)
Supplement: Supplementary file 1 [file DataSheet_1.doc]

Supplementary Material

Mapping knowledge landscapes and emerging trends of the biomarkers in melanoma: A bibliometric analysis from 2004 to 2022

Yantong Wan^1,†^, Junyi Shen^2,†^, Yinghao Hong^1,†^ , Jinghua Liu^1,*^, Tieliu Shi^4,5,*^, Junwei Cai^1,*^

**^1^** Guangdong Provincial Key Laboratory of Proteomics, Department of Pathophysiology, School of Basic Medical Sciences, Southern Medical University, Guangzhou, China

**^2^** The Second School of Clinical Medicine, Southern Medical University, Guangzhou, China

**^3^** The Center for Bioinformatics and Computational Biology, Shanghai Key Laboratory of Regulatory Biology, the Institute of Biomedical Sciences and School of Life Sciences, East China Normal University, Shanghai 200241, China

**^4^** Beijing Advanced Innovation Center for Big Data-Based Precision Medicine, Beihang University & Capital Medical University, Beijing 100083, China.

*** Correspondence:** Junwei Cai: cjw90107@smu.edu.cn; Tieliu Shi: tieliushi@yahoo.com; Jinghua Liu: liujhua@smu.edu.cn

**†** These authors contributed equally to this work.

# Supplementary Figures


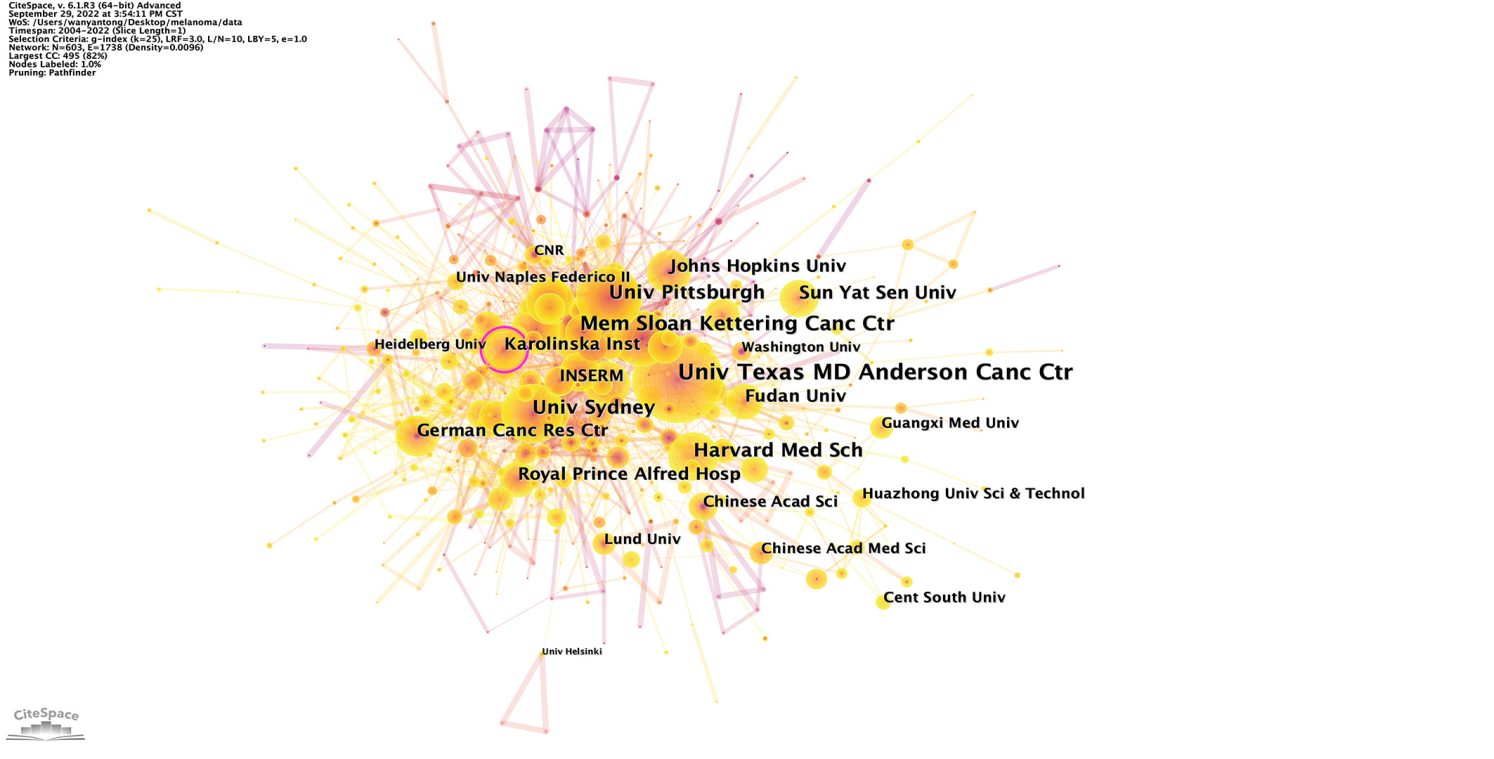


**Supplementary Figure 1.** Analysis of collaborative network visualization of institutions in CiteSpace. The size of the nodes indicates the co-occurrence frequencies, the links indicate the co-occurrence relationship, and the nodes with purple outer circles represent their higher centrality.
